# Nutrition, Diet and Healthy Aging

**DOI:** 10.3390/nu14010190

**Published:** 2021-12-31

**Authors:** Emiliana Giacomello, Luana Toniolo

**Affiliations:** 1 Department of Medicine, Surgery and Health Sciences, University of Trieste, 34149 Trieste, Italy; 2 Laboratory of Muscle Biophysics, Department of Biomedical Sciences, University of Padova, 35131 Padova, Italy

The current increase in life expectancy is confirmed by data from different sources (i.e., The World Population Prospects 2019 issued by the United Nations; https://population.un.org/wpp/ (accessed on 20 December 2021)), which predict that, in the near future, individuals who are over 65 and over 80 will be the fastest-growing portion of the population.

Although the increase in lifespan is a positive effect of the world’s improved sanitary, nutritional, and socioeconomic conditions, it is not free of complications. Actually, this improved lifespan is associated with an intensification of chronic diseases, such as cardiovascular disease, diabetes, cancer, sarcopenia, and degenerative disorders, which are grouped into what are called non-communicable diseases.

Aging is determined by the functional alteration of cell- and organ-related mechanisms that contribute to the functional decline of the individual. These alterations converge to a complex condition called frailty, where the individual experiences a loss of physical and psychological abilities, leading to a state of increased risk for adverse health outcomes in several pathologies. 

At present, the frailty index is a clinically evaluated method that can be used to assess the quality of aging, which depends on the individual’s exposition to different physical activities, nutritional regimens, and other socioeconomic characteristics. Therefore, frailty results in a modifiable condition that can be improved with affordable adjustments in lifestyle, such as following a nutritional regimen that meets the needs of aged people.

The aim of this Special Issue titled “Nutrition and Healthy Ageing” is to provide an overview on the connection between nutrition and aging, update knowledge on the mechanisms that are responsible for aging, and report on nutritional strategies that can be implemented to overcome age-related diseases.

This Special Issue collects 14 research articles and 4 reviews that span from studies in the population to basic research on experimental models. Overall, the collection touches themes such as dietary diversity, calorie restriction regimens, physical exercise, antiaging agents, social issues, and strategies to promote healthy aging. Attesting to the widespread interest in the correlation between nutrition and healthy aging, the current issue reports data on the correlation between diet and healthy aging from China, Italy, Korea, Malaysia, Mexico, Saudi Arabia, Spain, and the United States of America. 

The most marked message that emerges from this collection is the correlation between the frailty index and dietary diversity. Studies from different countries with very diverse nutritional habits show that aged individuals with a good diet variety score present a better frailty index [1,2,3,4,5,6,7,8,9,10]. Evidently, due to the diverse geographical origin of the data, it is difficult to infer what the best diet to avoid or to postpone the occurrence of frailty is. Moreover, as determined by the study of Avila and collaborators [11], other socioeconomic factors can increase the risk of malnutrition and can negatively influence the health status of aging people.

The maintenance of mental capacities is one important factor that characterizes healthy aging. As raised by work from Liu and collaborators [7] from and Zhang [2] and collaborators, the psychological conditions of aged people could also be correlated with dietary variety, and a high intake of carbohydrates and fat could be associated with memory impairment. 

Although we are far from identifying an ideal diet for a healthy aging, it can be summarized that adherence to Mediterranean diet for Europeans [5], the use of a balanced diet rich in vegetables in U.S. [3], and the general balanced equilibrium of vegetables, oil, fish, and meat in the diet seems to have a major role in maintaining a lower frailty index and in the prevention of the non-communicable diseases. 

The fact that aging is a multifaceted phenomenon that results from one or more failures at the molecular, cellular, physiologic, and functional levels, makes age-related diseases challenging therapeutic targets. Moreover, studies on aging and antiaging agents in humans can be difficult and can provide heterogeneous results due to the above-mentioned sanitary, geographical, and socioeconomic characteristics. In this issue, an example is provided by the review of Peladic and collaborators [12], which analyzes the literature on the role of probiotics on the modulation of inflammaging. The authors report heterogeneous evidence and come to the conclusion that probiotics have a limited effect on the modulation of low-grade inflammatory conditions in old individuals. 

Although research based on the use of experimental models has several hurdles [13], it represents an important resource to study the molecular mechanisms underlying aging, therefore helping in the search for potential targets. The use of these experimental models provides the opportunity to understand the mechanisms of action of micronutrients and to search for dietary regimens or molecules that can ameliorate the signs of aging or that can postpone aging. This Special Issue also provides a contribution to the broad field of antiaging strategies that have been tested in experimental models. Shen and collaborators show that attano-extracted tocotrienols improve the inflammatory and oxidative condition and the metabolism of macronutrients in obese mice [14]. Markovics and collaborators report that the anthocyanin-rich extract of sour cherry can improve the hyperglycemia-induced dysfunction of the endothelium in cultured human umbilical cord vein endothelial cells [15]. Martin and collaborators show that calorie restriction prevents the changes in the hypothalamic neuropeptides that are associated with obesity and metabolic dysregulation in rats [16].

In line with the sentence reported on the Pan American Health organization website, “Healthy aging is a continuous process of optimizing opportunities to maintain and improve physical and mental health, independence, and quality of life throughout the life course” (https://www.paho.org/en/healthy-aging (accessed on 20 December 2021)), prevention will have a central role in limiting age-related consequences. In this regard, Ottolini and collaborators [17] stress the influence of milk quality for feeding premature neonates on neurodevelopment. Additionally, as reported in the study protocol by Mohd Suffian and collaborators [18], there is an increasing interest in interventions aimed at promoting the prevention of frailty.

Being that the field of healthy aging is the subject of continuous modifications, future studies that are aimed at elucidating the processes that are involved in aging and their correlation with nutrition would be helpful to improve the diet quality in the population to prevent or reduce age-related complications. 

## Data Availability

Not applicable.
